# Complications During the Recovery Period After Pilonidal Sinus Surgery

**DOI:** 10.7759/cureus.4501

**Published:** 2019-04-19

**Authors:** Murat Kanlioz, Ugur Ekici

**Affiliations:** 1 General Surgery, Ministry of Health, Ankara, TUR; 2 General Surgery, Istanbul Gelişim University, Istanbul, TUR

**Keywords:** pilonidal sinus, postoperative complication, increased cost, limberg flap, limberg flap, surgical complications, spinal anesthesia, wound infection, abcess, seroma

## Abstract

Introduction

The current study aims to identify the complications that occur during the postoperative three-week period, which is considered the period of recovery in patients who undergo pilonidal sinus surgery. This identification of complications will help reduce morbidity and treatment costs and improve return to work.

Methods

This study included a total of 196 patients who underwent pilonidal sinus surgery by a combination of the resection and Limberg flap techniques under spinal anesthesia between the years 2012 and 2016. The postoperative three-week period was examined as the hospital stay period and the post-discharge period. The complications were classified into two groups: surgical and anesthesia. Results were recorded and analyzed using the SPSS statistical software (IBM Corp., Armonk, NY, US). p˂0.05 was considered significant.

Results

The female-to-male ratio of the patients was 1:4, whereas their average age was 24.15 years, the median age was 22 years, and the average body mass index was 24.79 kg/m². The average lengths of hospital stay in the postoperative period were 1.10, 2.15, and 3.95 days in patients without complications, all of the patients studied, and patients with complications, respectively. The difference between the groups was found statistically significant (p˂0.001).

Conclusion

Anesthesia-related and surgical complications were prominent in the postoperative hospital stay and post-discharge periods, respectively. The average length of hospital stay in patients with complications during hospital stay was found to be 3.59 times higher than those without complications. The difference between these two categories was statistically significant (p˂0.001).

## Introduction

Pilonidal sinus was first described by Hodges in 1880 [1]. This is an infective disease seen in the natal cleft and sacrococcygeal region. In the natal cleft, it is characterized by sinus openings in the midline, five cm away from the anus [2]. Whether the pilonidal sinus is congenital or acquired has been a matter of debate for a long time. With respect to this, many theories have been proposed so far. Today, however, it is generally accepted that this is an acquired disease [3-4]. The process of developing pilonidal sinus begins with the exposure of the pilosebaceous glands in the natal cleft to sex hormones during puberty. It is seen at an earlier age in women, most frequently at the age of 20-25. The female-to-male ratio varies between 1:3 and 1:5. It is more commonly seen in those who have a dark skin tone, are overweight, have a lot of body hair, and have oily skin [5]. The treatment procedures used are curettage, phenol applications, laser therapy, and surgery. The surgery involves open resection followed by primary closure and flap closure. This still remains the most accepted treatment procedure for pilonidal sinus [6]. Micro-invasive methods and endoscopic applications are also used [7-8].

## Materials and methods

This retrospective study included a total of 196 patients who underwent resection followed by Limberg flap repair under spinal anesthesia for pilonidal sinus disease between 2012 and 2016 at Malatya State Hospital; all these patients' records were complete. The patients' demographic data, such as age, gender, height, weight, length of hospital stay, whether the disease was primary or recurrent pilonidal sinus, and postoperative complications, were recorded in line with the planned study program. The developments over three weeks, which is considered as the postoperative recovery period, were followed and analyzed. In this follow-up, the postoperative three-week period was classified into two groups, these included: the hospital stay and the post-discharge period.

The complications were classified into two groups, namely, surgical complications and anesthesia complications, and then recorded. Following the classification of the complications into surgical and anesthesia complications, they were grouped into subgroups for the purposes of elaboration. The surgical complications were analyzed under five subgroups, including wound formation, wound site infection, and the formation of an abscess and/or hematoma under the flap, flap necrosis, and others. The spinal anesthesia-related complications were, however, analyzed under six subgroups, namely, headache, nausea-vomiting, low back pain, hypotensive symptoms, globe vesicle, and other. The results were recorded and analyzed using the SPSS statistical software (IBM Corp., Armonk, NY, US). The differences between the groups were evaluated on the basis of Pearson's chi-square test. p˂0.05 was considered significant.

## Results

Of the patients, 40 (20.4%) were female and 156 (79.6%) were male, whereas the female-to-male ratio was approximately 1:4, the average age was 24.15 years, the median age was 22 years, and the average body mass index (BMI) was 24.79 kg/m². Of the 196 patients, 103 (52.5%) were classified as overweight or obese according to their BMI. Of the patients, 170 (86.7%) were primary cases and 26 (13.3%) were relapsed cases. The average lengths of hospital stay in the postoperative period were approximately one, two, and four days in patients without complications, all the patients studied, and those patients with complications, respectively (Table 1). During the postoperative hospital stay, 72 (37%) patients were found to have complications, of whom 10 (14%) had surgical complications, 47 (65%) had only anesthesia complications, and 15 (21%) had both surgical and anesthesia complications. This result showed that one out of every three patients undergoing surgery developed complications in the hospital stay period.

**Table 1:** Duration of postoperative hospital stay according to the presence of surgical and spinal anesthesia-related complications n: Number of patients, **: 15 patients had both surgical and anesthesia-related complications, *: p˂0.001

	Duration of Postoperative Hospital Stay (Day)
General (n:196)	2.15
Those without postoperative complications (n:124, 63%)	1.1
Those with postoperative complications (n:72, 37%)**	3.95*

Of the anesthesia complications developed in 62 (32%) patients during the hospital stay (47 (76%) alone and 15 (24%) with surgical complications), 10 (16%) were in females and 52 (84%) were in males, and the female-to-male ratio was about 1:5. The most common anesthesia complications in this period were found to be headache in 28 (45%) patients, low back pain in 20 (32%) patients, and globe vesicle in 11 (18%) patients, respectively. All of those who developed globe vesicle were male patients (Table 2). Of the patients studied, the female-to-male ratio was 1:4, whereas the female-to-male ratio of the anesthesia complications that occurred during the postoperative hospital stay was found to be 1:5. Anesthesia-related complications were statistically significantly higher in males than in females (p˂0.01).

**Table 2:** Spinal anesthesia-related complications in the postoperative hospital stay in all patients *: p˂0.01

		None	Headache	Low Back Pain	Headache, Nausea, & Vomiting	Globe Vesicle	
Gender	Female	30	4	5	1	0	40
	Male	104	24 *	15 *	2	11 *	156
Total		134	28	20	3	11	196

Of the surgery-related complications seen in 25 (13%) patients during the hospital stay, four were in female patients and 21 (84%) were in male patients, and the rate of female-to-male complication was, again, about 1:5, namely at the same level as for spinal anesthesia-related complications. The most common surgical complications in the hospital stay period were wound site infection and seroma accumulation in 23 (92%) patients (Table 3). The female-to-male ratio of the patients in the study group was 1:4, whereas the female-to-male ratio of the surgery-related complications that occurred during the postoperative hospital stay was found to be 1:5. The difference between these ratios was statistically significant (p˂0.01). Surgery-related complications were also higher in males than in females in the postoperative hospital stay period.

**Table 3:** Surgical complications in the postoperative hospital stay period *: p ˂0.01

		None	Local Wound Site Complications	Other	
Gender	Female	36	3	1	40
	Male	135	20 *	1	156
Total		171	23	2	196

In the post-discharge period, however, a total of 59 (30.1%) patients were found to have complications. Of these, 47 (80%) had surgical complications, eight (14%) had anesthesia complications, and four (6%) had both surgical and anesthesia complications. Of 59 patients who developed complications in the post-discharge period, 41 (69%) had had surgical and anesthesia-related complications in the postoperative hospital stay period too. The number of patients who had complications only in the post-discharge period was found to be 18 (31%). Following their discharge from the hospital, two patients were readmitted due to post-spinal headache, nausea/vomiting, or hypotensive complaints and were given medical support. The other 57 (97%) patients were treated as outpatients. Most often, the complications encountered in the post-discharge period were wound site infection in 28 (47%) patients, abscess and seroma accumulation in 14 (24%) patients, and headache in 10 (17%) patients. Partial superficial flap necrosis occurred only in one patient (Table 4). Surgery-related complications were also statistically significantly higher in males than in females in the post-discharge period (p<0.01).

**Table 4:** Complications in the post-discharge period *: p<0.01

		None	Wound Site Infection	Wound Formation	Abscess & Seroma	Flap Necrosis	Post-Spinal Anesthesia Complication	Other	
Gender	Female	28	5	2	1	0	4	0	40
	Male	109	23 *	2	13 *	1	6	2	156
Total		137	28	4	14	1	10	2	196

## Discussion

The demographic data, such as the age and gender, of the studied patients were found to be close to the averages in the literature. Whereas our study had a female/male ratio of 1:4, it was 1:17 in the Onder et al. study and 1:8 in that by Anderson et al. [9-10]. The reason why we identified the postoperative follow-up period as three weeks was that the recovery period is considered three weeks, on average, in many publications and in line with our observations as well. Also, in our study, the average recovery time was determined as about three weeks. In some studies, however, the recovery periods are reported to be between 13 and 30 days [10-11].

It was observed that the most important factor determining the length of postoperative hospital stay was the complications developed. The average lengths of hospital stay in 124 patients who did not develop complications, in 196 patients studied, and in 72 (37%) patients who developed complications in the postoperative hospital stay period were found to be one, two, and four days, respectively (Table 1). The difference in the lengths of hospital stay between the groups was statistically significant. In the group with complications, it was longer (p˂0.001). It is seen that anesthesia-related complications are higher the period of postoperative hospital stay (Table 2). In our literature review, we found few studies on complications in the postoperative recovery period. In their study, Al-Khayat et al. studied the postoperative infection rate, which was found to be 12.8% [12]. In our study, however, the number of patients with infection was 23 (11.1%) in the hospital stay period followed by five more patients in the post-discharge period, which resulted in wound site infection in a total of 28 (14.2%) patients.

It was also found that both complication types, namely, the surgery- and anesthesia-related complications, in the postoperative hospital stay period were more common in male patients. Whereas the female-to-male ratio was 1:4 in our study, that of the complications developed was found to be 1:5 (female:male). When the ratio of men among all patients and the ratio of men among patients with complications were compared, the incidence of males was significantly higher in the group with complications (p˂0.01). In their study, Onder et al. reported that the rate of both complications was equal in both sexes [9]. However, in this study, there was no distinction between the hospitalization period and post-discharge complications.

In the post-discharge period, a total of 59 patients was found to have developed complications, of whom 12 were female and 47 were male. The female-to-male ratio was found to be about 1:4, which is at the same level as the female-to-male ratio in our study. Whereas the complication rate in females during the post-discharge period was 30%, it was 30.12% in male patients. No statistical significance was found in terms of the ratio of men and women developing complications in the post-discharge period.

Our study revealed the causes of complications occurring during the postoperative hospital stay and post-discharge periods. The most remarkable cases in the hospital stay period are the excess number of anesthesia-related complications and the prominence of those complications in male patients. The difference between the groups with and without complications is 3.59 times in terms of the average days of hospital stay. The repair procedure with the Limberg flap is still considered the most effective and safe method for the treatment of pilonidal sinus [13]. Given the complications, increased costs, and workforce loss, however, endoscopic treatments may be an alternative [14]. Recent methods, such as de-epithelization, etc. may also be considered as complementary therapy [15].

## Conclusions

After pilonidal sinus surgery, spinal anesthesia-related complications were prominent in the postoperative hospital stay (with a predominance in male patients) while surgical complications were prominent in the post-discharge period. These complications result in an increase in cost and a longer period before return to work. This study suggests that careful spinal anesthesia application, early medication for spinal anesthesia complications, and safe surgical practices will reduce complications post pilonidal sinus surgery.
